# Genome Sequence of a Novel Reassortant H3N6 Avian Influenza Virus from Domestic Mallard Ducks in Eastern China

**DOI:** 10.1128/genomeA.00223-12

**Published:** 2013-04-11

**Authors:** Qunhui Li, Lei Zhong, Qingqing Zhao, Liang He, Zhiqiang Duan, Chaoyang Chen, Yuxin Chen, Min Gu, Xiaoquan Wang, Xiaowen Liu, Xiufan Liu

**Affiliations:** Animal Infectious Disease Laboratory, College of Veterinary Medicine, Yangzhou University, Yangzhou, Jiangsu, Chinaa;; Ministry of Education Key Lab for Avian Preventive Medicine, Yangzhou University, Yangzhou, Jiangsu, Chinab

## Abstract

Here, we report the complete genome sequence of an H3N6 avian influenza virus (AIV) isolated from domestic ducks in Jiangsu province of eastern China in 2010. Phylogenetic analysis showed that the H3N6 virus is a natural recombinant virus whose genes were derived from H3N8, H4N6, H6N6, H7N7, and H11N2 AIVs. This analysis will help to understand the molecular characteristics and evolution of the H3N6 influenza virus in eastern China.

## GENOME ANNOUNCEMENT

Avian influenza viruses (AIV) are members of the family *Orthomyxoviridae* and have been shown to have 17 hemagglutinin (HA) and 10 neuraminidase (NA) subtypes (1, 2). Some of these subtypes have been transmitted to domestic poultry, causing severe or mild diseases, and domestic ducks play an important role in the transmission of influenza virus from wild aquatic birds to terrestrial poultry (3). However, H3 avian influenza viruses are one of the most frequently isolated subtypes from feral ducks and also the major subtype that causes human disease (4, 5). In addition, previous studies demonstrated that some novel H3N6 subtype viruses were reassortants between highly pathogenic H7 and H5 viruses isolated in Eurasia (6). Therefore, it is important to enhance the surveillance of H3 AIVs for understanding the genesis and emergence of novel reassortants with pandemic potential.

In this study, strain A/duck/Jiangsu/4/2010 (H3N6) was isolated from apparently healthy domestic mallard ducks in the Jiangsu province of eastern China. The complete genomic sequence was determined by reverse transcription-PCR (RT-PCR) using a universal primer set (7). The amplification products were purified and sequenced on an ABI 3730 capillary DNA-sequencing instrument. MEGA5.0 software was used to analyze the genomic sequences.

The complete genomic segments include polymerase basic 2 (PB2), PB1, polymerase acidic (PA), HA, nucleoprotein (NP), NA, matrix (M), and nonstructural (NS) genes, with full lengths of 2,341, 2,341, 2,233, 1,765, 1,565, 1,464, 1,027, and 890 nucleotides, respectively. The amino acid sequence at the cleavage site in the HA molecule is PEKQTR↓G, which is characteristic of low-pathogenic AIV. Analysis of potential glycosylation sites of the isolate revealed that there were 6 potential *N*-linked glycosylation sites in HA (positions 8, 22, 38, 165, 285, and 483), while there were 9 in NA (positions 51, 54, 62, 67, 70, 86, 146, 201, and 402). Furthermore, there were no changes in the length of the NA stalk region and the NS1 protein.

Phylogenetic analysis showed that the nucleotide sequence identity of the HA gene with that of the H3N8 isolate A/duck/Beijing/40/04 was 99%. The nucleotide sequence identities of the PB2, PB1, PA, and M genes with those of the H6N6 isolate A/duck/Jiangsu/022/2009 (H6N6) were all 99%. The nucleotide sequence identity of the NS gene with that of the isolate A/mallard/Korea/GH171/2007 (H7N7) was 98%. The nucleotide sequence identity of the NA gene with that of the isolate A/chicken/India/WB-NIV101006/2009 (H4N6) was 99%. In addition, the NP gene was most closely related to that of the isolate A/spotbill duck/Xuyi/6/2005 (H11N2), with which it shares 99% nucleotide homology. Thus, the H3N6 virus proved to be a novel multiple-gene reassortant AIV whose genes were derived from H3N8, H4N6, H6N6, H7N7, and H11N2.

Therefore, the genome information of A/duck/Jiangsu/4/2010 (H3N6) will help in analyses of the epidemiology and evolutionary characteristics of AIV in domestic ducks in China.

### Nucleotide sequence accession numbers. 

The complete genomic sequence of A/duck/Jiangsu/4/2010 (H3N6) was deposited in GenBank under the accession no. KC261674 to KC261681.
